# Reference standards for the 6-min walk test in Croatian older adults

**DOI:** 10.3389/fphys.2023.1226585

**Published:** 2023-08-04

**Authors:** Peter Sagat

**Affiliations:** GSD/Health and Physical Education Department, Sport Sciences and Diagnostics Research Group, Prince Sultan University, Riyadh, Saudi Arabia

**Keywords:** functional capacity, standards, older adults, charts, differences

## Abstract

**Introduction:** The 6-min walk test (6MWT) is commonly used to assess the level of functional capacity of individuals with respiratory diseases. Although previous evidence has provided reference standards for the 6MWT in unhealthy older adults, no data have been provided for the Croatian healthy older populations. Therefore, the main purposes of the study were to define sex- and age-specific references for the 6MWT in older adults.

**Methods:** Six-hundred and forty-three older adults (260 men and 383 women) aged 60–80 years were recruited in this observational cross-sectional study. The participants were instructed to walk the maximal distance possible for 6 min. The main outcome was the final score in the 6MWT conducted at a 30-m straight corridor.

**Results:** Men exhibited longer walking distance, compared to women (678.3 ± 59.1 vs. 653.8 ± 49.9 m, *p* < 0.001) and younger men and women performed better, than their older counterparts (*p* < 0.001). However, the sex*age interaction effect showed no significant differences between men and women in the same age range (*p* = 0.865).

**Discussion:** This is the first study with the purpose of providing reference standards for the 6MWT in a large sample of Croatian older adults. Older men and women in lower percentiles may be treated as a “risky group” of individuals with a special attention of implementing interventions to enhance the performance.

## Introduction

The 6-min walk test (6MWT) is considered a reliable and valid field-based exercise test to assess the level of functional capacity at submaximal level (Solway et al., 2001; ATS Committee on Proficiency Standards for Clinical Pulmonary Function Laboratories, 2002; Casanova et al., 2011; Makni et al., 2020). The purpose of the test is to walk at self-paced speed over the period of 6 min (ATS Committee on Proficiency Standards for Clinical Pulmonary Function Laboratories, 2002). It assesses prognosis and evaluates a specific response to different treatments, especially in individuals with respiratory diseases (Enright et al., 2003; Holland et al., 2014; Singh et al., 2014). Lower performance in the 6MWT has been associated with premature mortality in patients suffering from chronic respiratory (Pinto-Plata et al., 2004; Casanova et al., 2005) and cardiovascular (Miyamoto et al., 2000) diseases.

Although the accessibility and practical implications of the 6MWT have been well-documented (ATS Committee on Proficiency Standards for Clinical Pulmonary Function Laboratories, 2002), previous research has been mainly concentrated on using the 6MWT for intervention outcomes (Salzman, 2009). To be able to measure the effectiveness of a certain intervention, the American Thoracic Society (ATS) has published the standardized procedures for undertaking the 6MWT, with a special intention to establish reference values for different populations (ATS Committee on Proficiency Standards for Clinical Pulmonary Function Laboratories, 2002; Casanova et al., 2011). By generating such data, health policymakers may be able to screen, monitor and track changes in the 6MWT at individual or group level. During the past two decades, several studies have elaborated the 6MWT reference standards. The majority of them have been conducted in the USA (Enright and Sherrill, 1998; Enright et al., 2003; Cote et al., 2008), Canada (Gibbons et al., 2001; Hill et al., 2011), Brazil (Iwama et al., 2009; Dourado et al., 2011; Soaresa and Pereira, 2011; Britto et al., 2013; Korn et al., 2014), Chile (Osses et al., 2010), Australia (Camarri et al., 2006; Jenkins et al., 2009), Asia (Poh et al., 2006; Teramoto et al., 2006; Alameri et al., 2009; Kim et al., 2014; Palaniappan Ramanathan and Chandrasekaran, 2014; Zou et al., 2017), Africa (Ben Saad et al., 2009) and Europe (Troosters et al., 1999; Chetta et al., 2006; Casanova et al., 2011; Beekman et al., 2014; Duncan et al., 2015; Oliveira et al., 2019; Cazzoletti et al., 2022). Although an effort has been made to establish normative data for the 6MWT, there has been a relatively great heterogeneity between the studies in terms of walking distance and sociodemographic, anthropometric and cross-cultural differences, which can affect the test performance (ATS Committee on Proficiency Standards for Clinical Pulmonary Function Laboratories, 2002). To the best of our knowledge, no reference standards have been obtained for the Croatian population. A recent population-based study conducted among a national representative Croatian sample has shown the low prevalence of individuals engaging in muscle-strengthening activities, being the lowest in the 65+ year-olds (Radašević et al., 2021). Since the performance in the 6MWT is a measure of functional status (Enright et al., 2003), it is necessary to develop normative charts and cut-of points.

Therefore, the main purpose of the study was to create sex- and age-specific reference standards for the 6MWT in Croatian older adults aged 60–80 years.

## Materials and methods

### Study participants and design

**FIGURE 1:**
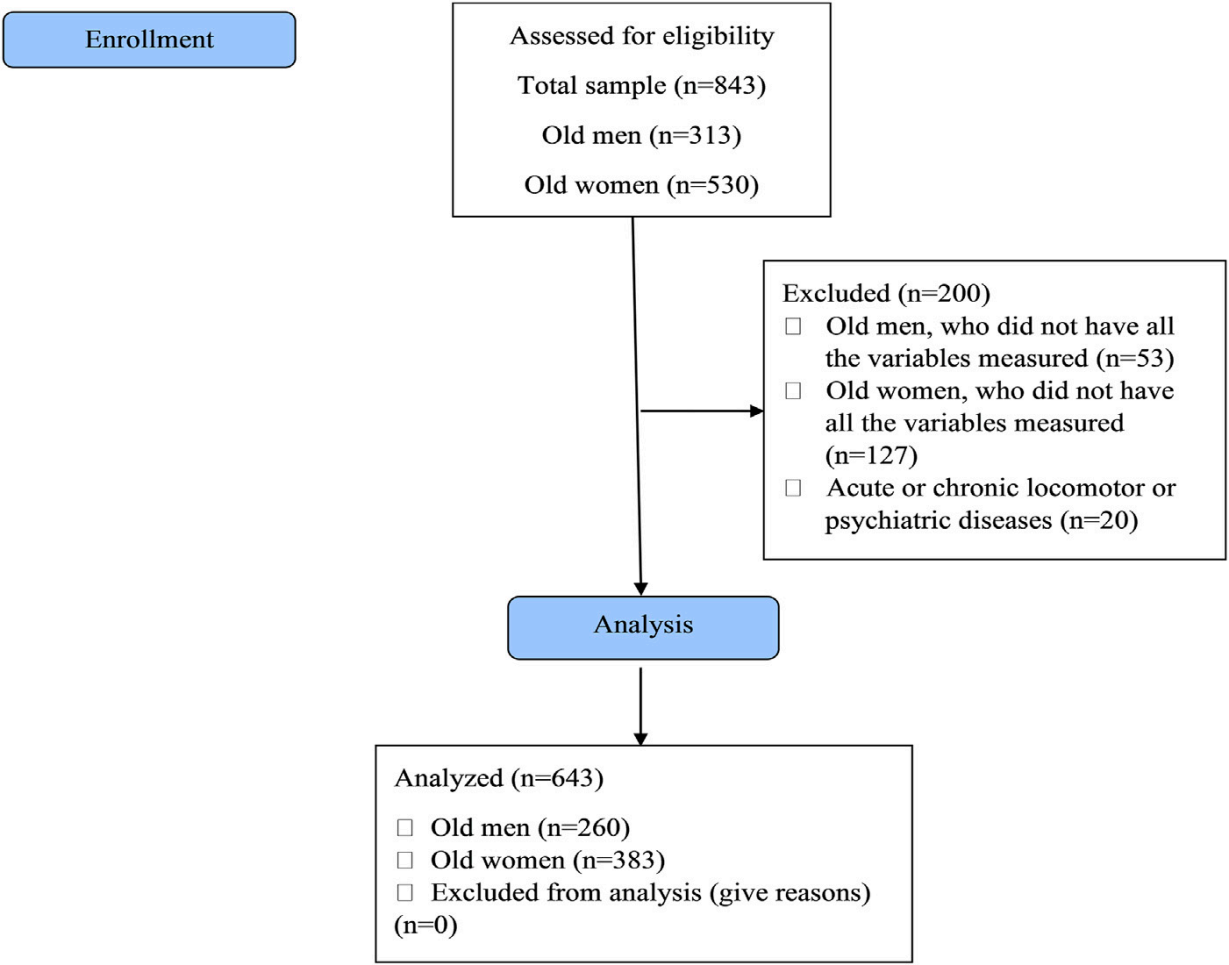
A flow chart diagram.

In this observational prospective cross-sectional study, we recruited 843 participants, who were part of a single rehabilitation center study in the “Lipik” county near the city of Zagreb from 2020 to 2022. The general aim of the project was to explore the lifestyle habits of apparently healthy older adults, who went through annual physical and mental systematic examinations. The inclusion criteria for participation in the study included: 1) being without chronic diseases, which included chronic heart disease, rheumatic arthritis, chronic kidney disease, stroke, cancer and chronic obstructive pulmonary disease, 2) the absence of a serious physical or mental illness, and 3) being tested for s the 6MWT. Of these, 200 did not meet the inclusion criteria; i.e., 180 participants did not complete the 6MWT, due to health issues during testing (the shortage of breath, dizziness, chest pain) and 20 had acute or chronic locomotor or psychiatric disease. After re-analysis, 643 men (mean age: 67.4 ± 5.5 years) and women (66.9 ± 5.2 years) met the inclusion criteria and were included in the final analysis. A detailed recruitment of the participants is shown in Figure 1. Before data collection started, all participants were informed about the aim, hypotheses and methodology of the study. The participants were ensured confidentiality and informed that their participation was voluntary, and that they had the right to withdraw at any time. Data collection was done in the same setting by the same experimental team, minimizing the effect of a measurement error. For the purpose of this study, the main components that needed to be measured to create reference standards were sex, age, height, weight and the 6MWT. All participants have read and signed the informed consent forms. The Ethical Committee of The Home of War Veterans approved the study (Ethical code number: 2022/4).

### The 6MWT

The test was conducted using a 30-m straight corridor with a flat, firm ground and with two cones placed at each end of the course. We followed the testing procedure from the ATS Committee guidelines (ATS Committee on Proficiency Standards for Clinical Pulmonary Function Laboratories, 2002). All participants were instructed to walk the maximal distance possible for 6 min. The final score was expressed in distance covered in meters (m) during a 6-min period. Height and weight were objectively measured using portable stadiometer and digital scale with a precision of 0.1 mm and 0.1 kg. Body-mass index was calculated using the following formula: weight (kg)/height (m^2^). Age was self-reported.

### Statistical analysis

Basic descriptive statistics are presented as mean and standard deviation (SD) and as median and 25th—75th interquartile range (IQR). The Kolmogorov–Smirnov tests showed that data were normally distributed. Sex and age differences were calculated by using analysis of variance (ANOVA) with *post hoc* comparison test between the groups. To calculate correlations between all the study variables, we used Pearson coefficient of correlation (*r*). Finally, the regression equation to predict the 6MWT was performed. The independent variables included in the equation were sex, age, height and weight. Coefficients of correlation (*R*) and determination (*R* ^*2*^) were used to establish the association and the % of variance explained for the dependent variable. All the assumption, including the level of Leven’s test of homogeneity, normal population distribution and data independency were met. All analyses were performed in Statistical Packages for Social Sciences version 24. (SPSS Inc., Chicago, Illinois, United States).

## Results

Basic descriptive statistics are presented in Table 1. Men were taller, heavier and had higher body mass index (BMI) values, compared to women (*p* < 0.05). Women exhibited lower distance covered in 6 min, compared to men. The 6MWT was significantly correlated with age (*r* = −0.72, *p* < 0.001), height (*r* = 0.28, *p* < 0.001), weight (*r* = −0.23, *p* < 0.001) and body-mass index (BMI; *r* = −0.47, *p* < 0.001).

**TABLE 1:** Basic descriptive statistics of the study participants, stratified by sex (*N* = 643).

Study variables	Men (*N* = 260)	Women (*N* = 383)	ES	*P* for sex
	Mean (SD)	Mean (SD)		
Age (years)	67.4 (5.5)	66.9 (5.2)	0.09	0.160
Height (cm)	172.9 (5.0)	161.1 (6.0)	2.14	<0.001
Weight (kg)	84.0 (10.3)	70.0 (12.1)	1.25	<0.001
BMI (kg/m^2^)	27.7 (3.3)	26.9 (4.2)	0.21	0.027
The 6MWT (m)	678.3 (59.1)	653.8 (49.9)	0.45	<0.001

*p* < 0.05.

**FIGURE 2:**
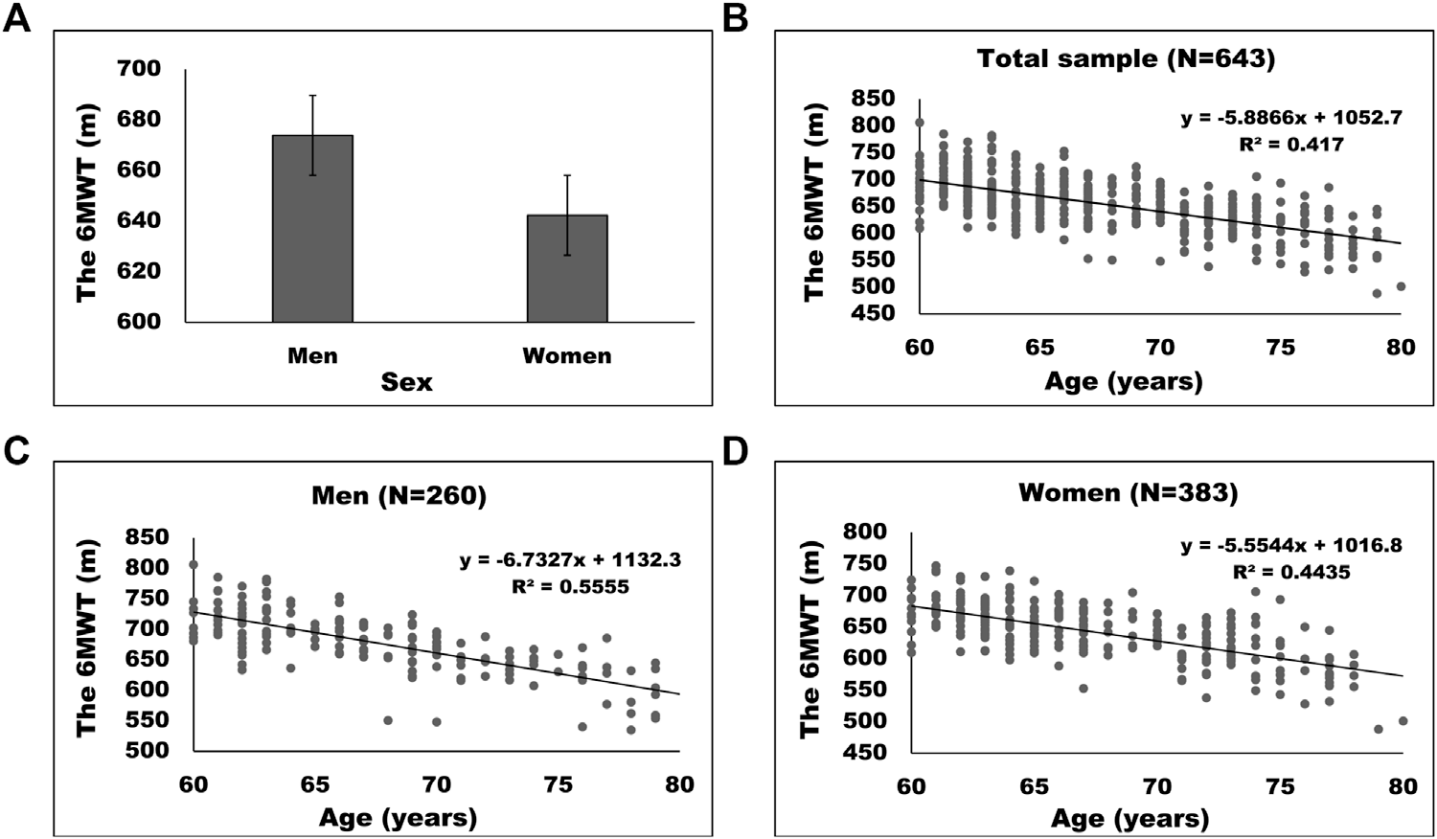
The 6MWT in men and women **(A)**, along with the association between the 6MWT and age in total sample **(B)**, in men **(C)** and in women **(D)**.

Figure 2 shows the 6MWT in men and women (A), along with the association between the 6MWT and age in total sample (B), in men (C) and in women (D). Normative values for the 6MWT are presented in Table 2. Men performed better than women and younger men and women exhibited better walking distance in the 6MWT, compared to their older counterparts. The interaction effect between sex and age was not statistically significant, pointing out that the main effect of age was not modified by sex and *vice versa*. More detailed statistical analyses for the 6MWT can be found in Table 3.

**TABLE 2:** Normative data for the 6MWT (m) and the peak VO_2_ (mL/kg/min), stratified by sex and age.

Study variable	Sex	Age	Mean	SD	Median	IQR (25th—75th^)^
The 6MWT	Men	60–64 years	714.7	37.0	714.0	691.8–740.6
		65–69 years	681.4	35.0	686.8	660.4–706.5
		70–74 years	650.7	29.4	652.7	637.3–673.1
		75–80 years	610.7	40.2	622.6	576.9–637.0
		Total	673.9	58.7	679.0	643.1–709.2
	Women	60–64 years	670.3	32.0	669.8	651.2–690.8
		65–69 years	649.9	30.0	653.1	624.2–674.5
		70–74 years	619.5	33.1	620.7	598.5–643.5
		75–80 years	583.6	42.9	581.2	560.6–604.1
		Total	642.3	48.0	649.4	616.0–673.9

**TABLE 3:** Main effects for the 6MWT and peak VO_2_ in the study participants, (*N* = 643).

Study variables	Total sample (*N* = 643)	Main effects		
	Mean (SD)/Median	Sex; *F*-value (*p-*value; eta squared)	Age; *F*-value (*p-*value; eta squared)	Sex * age; *F*-value (*p-*value; eta squared)
The 6MWT (m)	654.3 (54.5)/657.8	88.65 (<0.001; 0.17)	129.15 (<0.001; 0.47)	1.42 (0.236; 0.01)

Table 4 shows a set of regression equations to predict the 6MWT. In Model 1, sex explained 8.0% of the variance in the 6MWT (*R* = 0.29; *R* ^*2*^ = 0.080; *SEE* = 52.4 m; *p* < 0.001). When age was added to sex in Model 2, the model explained 54.3% of the variance in the 6MWT (*R* = 0.74; *R* ^*2*^ = 0.543; *SEE* = 33.3 m; *p* < 0.001). When the equation was adjusted for sex, age and height in Model 3, the model explained 75.7% of the variance in the 6MWT (*R* = 0.87; *R* ^*2*^ = 0.757; *SEE* = 24.3 m; *p* < 0.001). In the final model (Model 4), sex, age, height and weight were entered simultaneously and explained 88.4% of the variance in the 6MWT (*R* = 0.94; *R* ^*2*^ = 0.884, *SEE* = 16.8 m, *p* < 0.001).

**TABLE 4:** Simple regression equations to predict the level of the 6MWT based on sex, age, height and weight, (*N* = 643).

Study variables	Unstandardized estimates; 95% CI (*P*—value*)	Regression equation for the 6MWT
Model 1		
Constant (intercept)	705.56; 688.66 to 722.46 (<0.001)	705.56–31.65 * sex (1—men; 2—women)
Sex (1—men; 2—women)	−31.65; −41.65 to −21.65 (<0.001)	
Model 2		
Constant (intercept)	1,121.89; 1,081.15 to 1,162.62 (<0.001)	1,121.89—35.98 * sex (1—men; 2—women)—6.05 * age (years)
Sex (1—men; 2—women)	−35.98; −42.35 to −29.06 (<0.001)	
Age (years)	−6.05; −6.62 to −5.47 (<0.001)	
Model 3		
Constant (intercept)	394.58; 315.90 to 472.26 (<0.001)	394.58 + 11.44 * sex (1—men; 2—women)—5.66 * age (years) + 3.77 * height (cm)
Sex (1—men; 2—women)	11.44; 4.77 to 18.10 (<0.001)	
Age (years)	−5.66; −6.08 to −5.24 (<0.001)	
Height (cm)	3.77; 3.39 to 4.15 (<0.001)	
Model 4		
Constant (intercept)	308.16; 253.25 to 363.09 (<0.001)	308.16 + 5.59 * sex (1—men; 2—women)—5.55 * age (years) + 5.05 * height (cm)—1.65 * weight (kg)
Sex (1—men; 2—women)	5.59; 0.96 to 10.23 (0.018)	
Age (years)	−5.55; −5.85 to −5.26 (<0.001)	
Height (cm)	5.05; 4.77 to 5.34 (<0.001)	
Weight (kg)	−1.65; −1.80 to −1.50 (<0.001)	

## Discussion

The main purpose of the study was to create sex- and age-specific reference standards for the 6MWT in older adults aged 60–80 years. Our main findings are: 1) men performed better, compared to women, 2) younger men and women exhibited larger walking distance, compared to their older counterparts, and 3) non-significant sex*age interaction is observed.

To the best of our knowledge, this is the first study conducted in Croatia and one of the few examining reference values for the 6MWT in a relatively large sample of older adults. The median value obtained in this study was around 650 m for men and women, which corresponds to findings of previous research conducted in different nationalities (Troosters et al., 1999; Gibbons et al., 2001; Camarri et al., 2006; Iwama et al., 2009; Jenkins et al., 2009; Hill et al., 2011; Duncan et al., 2015; Zou et al., 2017; Oliveira et al., 2019). In specific, the distance walked over a 6 min period in these studies ranged between 600 and 650 m in Brazil (Iwama et al., 2009; Dourado et al., 2011; Soaresa and Pereira, 2011; Britto et al., 2013; Korn et al., 2014), Africa (Ben Saad et al., 2009) and Europe (Troosters et al., 1999; Chetta et al., 2006; Casanova et al., 2011; Beekman et al., 2014; Duncan et al., 2015; Oliveira et al., 2019; Cazzoletti et al., 2022). Other studies have reported somewhat smaller values, ranging from ≈350 m (Enright et al., 2003) to <600 m (Poh et al., 2006; Alameri et al., 2009; Soaresa and Pereira, 2011; Britto et al., 2013; Kim et al., 2014), while the largest greatest values have been observed in Australian population of >650 m (Camarri et al., 2006; Masmoudi et al., 2008). The heterogeneity between the studies comes from differences in participant recruitment regarding the ratio between men and women, sample size and age range and test instructions given during the protocol (Teramoto et al., 2006). Although the ATS has highlighted the recommended protocol for undertaking the 6MWT (ATS Committee on Proficiency Standards for Clinical Pulmonary Function Laboratories, 2002), there has been a considerable variation between the walking distance covered by individuals. Thus, examining reference data should be center-specific, in order to compare and to generalize the findings between the centers.

In agreement with the literature, we found a significant difference between men and women for the average 6MWT, where men performed better, compared to women (Enright et al., 2003; Camarri et al., 2006; Casanova et al., 2011; Soaresa and Pereira, 2011; Cazzoletti et al., 2022). From a biological point of view, men are generally taller and have a greater skeletal muscle mass. This is not surprising, since our data showed a stronger correlation between the 6MWT and height in men than women (*r* = 0.65 vs. *r* = 0.60, *p* < 0.001). On the other hand, weight seems to be more pronounced to affect the distance travelled in women (*r* = −0.16, *p* = 0.009), compared to men (*r* = −0.11, *p* = 0.135), which is in line with previous studies (Cazzoletti et al., 2022). This would imply that heavier individuals walk shorter distances, because changes in weight affect different energy requirements and work performance (Oliveira et al., 2019). Moreover, higher BMI values were significantly correlated with shorter distance travelled in both men (*r* = −0.49, *p* < 0.001) and women (*r* = −0.46, *p* < 0.001), which highlights the importance of adjusting the 6MWT for height and weight in future research (Enright and Sherrill, 1998; Troosters et al., 1999; Casanova et al., 2011; Zou et al., 2017). With that in line, one previous study has used an allometric scaling of the 6MWT for height and body mass, pointing out that such approach leads to the least SD of differences and the smallest coefficient of variation (CoV), compared to previous regression equations for the 6MWT (Duncan et al., 2015).

We also found an age-related decline of the 6MWT, where younger men and women exhibited greater distances, compared to their older counterparts. Results of this study correspond to previous findings conducted among older adults (Gibbons et al., 2001; Enright et al., 2003; Hill et al., 2011). Studies have shown, that the level of testosterone starts to decrease at the age of 40 (Enright and Sherrill, 1998), which directly affects the progressive reduction in skeletal muscle mass and lower relative muscle strength and endurance. However, a declining trend does not show similar behavioral patterns in men and women; in men, muscle mass rapidly decreases between 50 and 60 years, while women experience an increase, but at lower pace (Ofenheimer et al., 2020). However, women experience a greater loss of muscle mass, compared to men at later stages of life. Indeed, a study conducted among a Croatian representative sample has shown, that the level of muscle-strengthening activities changes by age, with the steepest decline being observed for older individuals (Radašević et al., 2021). Since physical performance has been associated with physical activity (Stenholm et al., 2016), it is not surprising that lower levels of physical activity at old age may lead to poorer physical performance in the 6MWT.

Finally, we observed a non-significant sex*age interaction, which means that men and women in the same age group exhibited similar values in the 6MWT. Although the results showed that men and younger men and women had better values in the 6MWT, compared to women and their younger counterparts, when comparing men and women at the same age (60–64 years, 65–69 years, 70–74 years, and 75–81 years), similar rates of change between the groups were observed. The reason for a non-significant main effect may be explained by a different men/women ratio in a certain age group and insufficient statistical power of the sample to detect sex- and age-specific significant differences. The second mechanism includes the level of motivation for completing the test, where previous evidence suggests that the lack of adequate effort may have influenced the results (Casanova et al., 2011). Finally, a multiple regression analysis conducted on our sample showed that sex and age explained 54.3% of the variance in the 6MWT, which means that 46% remained unexplained by our model, which is in line with previous studies (Enright and Sherrill, 1998; Gibbons et al., 2001; Enright et al., 2003; Casanova et al., 2011). Thus, by including other physiological and psychological parameters into the model would probably suggest in different results.

It has been well-established, that the 6MWT represents a reliable and valid field-based test to assess functional capacity at submaximal level (Casanova et al., 2011). Although its applicability and usefulness have been studied in previous literature, most of research has been mainly conducted in populations with disorders or in postoperative stages (Miyamoto et al., 2000; Pinto-Plata et al., 2004; Casanova et al., 2005). Thus, by examining reference standards in apparently healthy older adults, one would be able to screen, monitor and track the distance travelled in the 6MWT.

## Limitations

This study is not without limitations. First, the design of the study was cross-sectional, and we were unable to determine the longitudinal changes in the 6MWT. Second, the sample used in this study did not incorporate a diverse selection of ethnicities (only Caucasian men and women were included). Third, we did not collect any information regarding physiological and psychological variables, such as blood samples, the level of motivation, physical activity, and body composition, which might be able to explain sex and age reference standards in a deeper level. Finally, the model was not cross-validated with other independent samples of older adults from other countries to determine the sensitivity properties of the findings.

## Conclusion

In summary, this is the first study aiming to develop reference standards for the 6MWT in Croatian older adults aged 60–80 years. Our newly developed references should serve in clinical settings; to screen for submaximal functional capacity, to detect those with “poor” performance in the 6MWT and to track an individual’s rank in a certain group. Therefore, individuals recognized as a “risky group” should be a target population for special policies and strategies aiming to enhance functional performance and prolong independent living.

## Data Availability

The raw data supporting the conclusion of this article will be made available by the authors, without undue reservation.

## Abbreviations

ANOVA, analysis of variance; ATS, the American Thoracic Statement; CoV, coefficient of variation; LMS, Lambda, Mu and Sigma; Peak oxygen uptake, peak VO_2_max; SD, standard deviation; 6MWT, the 6-min walk test; 95% CI, 95 percent confidence interval.
